# Metabolomic Profiling of Hepatitis B-Associated Liver Disease Progression: Chronic Hepatitis B, Cirrhosis, and Hepatocellular Carcinoma

**DOI:** 10.3390/metabo15080504

**Published:** 2025-07-29

**Authors:** Junsang Oh, Kei-Anne Garcia Baritugo, Jayoung Kim, Gyubin Park, Ki Jun Han, Sangheun Lee, Gi-Ho Sung

**Affiliations:** 1 Biomedical Institute of Mycological Resource, International St. Mary’s Hospital, College of Medicine, Catholic Kwandong University, Incheon 22711, Republic of Korea; 645098@ish.ac.kr (J.O.); 645099@ish.ac.kr (K.-A.G.B.); kjy7@ish.ac.kr (J.K.); 2 Department of Laboratory Medicine, International St. Mary’s Hospital, College of Medicine, Catholic Kwandong University, Incheon 22711, Republic of Korea; 3 Department of Biomedical Science, Graduate School, Catholic Kwandong University, Gangneung-si 25601, Republic of Korea; coby160307@cku.ac.kr; 4 Department of Internal Medicine, International St. Mary’s Hospital, College of Medicine, Catholic Kwandong University, Incheon 22711, Republic of Korea; 545818@ish.ac.kr; 5 Department of Convergence Science, College of Medicine, Catholic Kwandong University, Gangneung-si 25601, Republic of Korea

**Keywords:** hepatitis B virus, serum metabolites, metabolic pathways, metabolomics, multivariate analysis, ^1^H-NMR

## Abstract

**Background/Objective:** The hepatitis B virus (HBV) can cause chronic hepatitis B (CHB), which can rapidly progress into fatal liver cirrhosis (CHB-LC) and hepatocellular carcinoma (CHB-HCC). **Methods:** In this study, we investigated metabolites associated with distinct clinical stages of HBV infection for the identification of stage-specific serum metabolite biomarkers using ^1^H-NMR-based metabolomics. **Results:** A total of 64 serum metabolites were identified, among which six core discriminatory metabolites, namely isoleucine, tryptophan, histamine (for CHB), and pyruvate, TMAO, lactate (for CHB-HCC), were consistently significant across univariate and multivariate statistical analyses, including ANOVA with FDR, OPLS-DA, and VIP scoring. These metabolites were closely linked to key metabolic pathways, such as propanoate metabolism, pyruvate metabolism, and the Warburg effect. **Conclusions:** The findings suggest that these six core metabolites serve as potential stage-specific biomarkers for CHB, CHB-LC, and CHB-HCC, respectively, and offer a foundation for the future development of metabolomics-based diagnostic and therapeutic strategies.

## 1. Introduction

Hepatitis B virus (HBV) infection causes acute and chronic liver diseases and is one of the major life-threatening global health problems [1]. However, the early treatment of HBV infection for the prevention of exacerbation is problematic since it is initially asymptomatic. HBV symptoms, such as fatigue, abdominal pain and vomiting, only occur after 1-to-6 months of infection. By then, the afflicted patients are at a high risk for the development of fatal diseases associated with HBV infection, such as chronic hepatitis B (CHB) and CHB-associated liver cirrhosis (CHB-LC) and hepatocellular carcinoma (CHB-HCC) [2].

HBV infection directly affects the liver, which plays a crucial role in metabolism. The liver also serves as the first point of entry for metabolites produced by the gut microbiome. The pathology of HBV-associated liver disease can be analyzed and explained in terms of its effect on metabolites in liver–gut axis. Thus, the analysis of the metabolic serum profiles of affected patients can help identify differential metabolites and altered pathways that characterize the effect and relationship between HBV-associated liver disease and the liver–gut axis. The identification of differential metabolites and altered pathways can also be used to develop a diagnostic system that can differentiate between the asymptomatic stage of HBV infection and severe clinical stages (CHB, CHB-LC, and CHB-HCC). Furthermore, the identification and quantification of serum metabolites can be the basis for the development of appropriate nutritional and/or therapeutic treatments for affected patients.

Metabolomics is powerful omics technology used for the detection and identification of metabolites from biological samples. This approach commonly uses analytical instruments, such as nuclear magnetic resonance (NMR) spectrometers and integrated liquid or gas chromatography–mass spectrometry (LC-MS, GC-MS) [3]. The metabolomic approach with the analysis of the clinical stages of liver diseases can provide novel insights into understanding the correlation between stage-specific disease severity and metabolite identity and quantity [4]. Thus, the comprehensive identification and comparison of key metabolites and associated altered metabolic pathways at each clinical stage is important for the precise diagnosis of liver disease development. These identified biomarkers and/or patterns can be used to discriminate between each stage of the disease so that appropriate preventative or remedial therapy can be prescribed and applied in a clinical setting. So far, several studies regarding the metabolomics of liver disease have focused solely on nonalcoholic fatty liver disease (NAFLD) and nonalcoholic fatty hepatitis (NASH) instead of chronic viral liver disease caused by hepatitis B [5,6,7]. In addition, those studies have focused on the analysis of serum profiles from patients with advanced forms of liver disease. On the other hand, only a handful of studies have used different metabolomic approaches for the investigation and comparison of differential metabolites associated with clinical-stage-specific, HBV-associated liver disease: (1) LC quadrupole time-of-flight MS and GC quadrupole time-of-flight MS for CHB-HCC vs. CHB-HCC + LC [8]; (2) GC/MS for CHB-LC vs. CHB and CHB-HCC vs. CHB-LC [9]; (3) LC/MS for CHB-HCC vs. CHB [10]; (4) ^1^H-NMR for HCC vs. LC [11]; (5) NMR and LC/MS for HCC vs. LC [12]; and (6) GC time-of-flight MS and ultra-performance liquid chromatography–quadrupole time-of-flight spectrometry for CHB + LC vs. CHB + HCC + LC vs. HCC [13]. However, the metabolomics of HBV-associated liver disease at early clinical stages for the identification of differential metabolites and the development of biomarkers for accurate identification and discrimination between distinct clinical stages of CHB vs. CHB-LC vs. CHB-HCC is not yet standardized and/or applied for general clinical diagnostic practice.

Thus, in this study we comprehensively identified and compared metabolites present in serum profiles of patients grouped according to the diagnosed clinical stage of HBV infection (CHB, CHB-LC, CHB-HCC) by using the ^1^H-NMR approach. Univariate and multivariate statistical approaches were used for the identification and quantification of significant differential metabolites and the comparison of the metabolic profiles of affected patients to each clinical stage of HBV-associated liver disease. Correlation and enrichment analyses were also applied to analyze the relationships between the identified metabolites and their effect on associated metabolic pathways at each stage of HBV infection. The metabolomics-based approaches used in the present study were able to identify novel metabolite biomarkers, which can be used to discriminate between each stage of HBV-associated liver diseases. The results observed in this study provide new insights into understanding the metabolic reprogramming that occurs during the development of HBV-associated liver disease.

## 2. Materials and Methods

### 2.1. Patient Information and Data Collection

The subjects described in the present study were patients diagnosed with CHB, CHB-LC, and CHB-HCC treated between 2016 and 2020 in Catholic Kwandong University International St. Mary’s Hospital in the Republic of Korea. The patients were categorized based on their clinical stage of HBV disease. The patients were eligible for inclusion if they were 18 years of age or older, had serologically confirmed chronic HBV infection with positive hepatitis B surface antigens by immunologic testing, and had never received antiviral therapy for HBV prior to enrollment. Eligible individuals were classified into three groups according to their clinical stage of HBV disease: (1) patients with CHB without any evidence of cirrhosis or malignancy; (2) patients with compensated liver cirrhosis due to CHB-LC, confirmed by abdominal ultrasonography and transient elastography, and without HCC; and (3) patients with CHB-HCC, diagnosed by histopathology and/or characteristic imaging findings on multi-slice computed tomography or dynamic contrast-enhanced magnetic resonance imaging. Inclusion also required the availability of comprehensive clinical and laboratory data and stored serum samples suitable for metabolomic analysis. Patients were excluded if they had evidence of other chronic liver diseases, including alcoholic liver disease, autoimmune hepatitis, drug-induced liver injury, Wilson’s disease, hemochromatosis, or biliary tract disease. Additional exclusion criteria included a history of significant alcohol consumption (>30 g/day for men and >20 g/day for women); decompensated cirrhosis, defined by the presence of moderate-to-severe ascites, overt hepatic encephalopathy, prior variceal bleeding, or Child–Pugh class B or C; severe renal impairment (estimated glomerular filtration rate < 30 mL/min/1.73 m^2^) or a need for dialysis; and the use of medications or interventions known to affect metabolic profiles, such as corticosteroids or immunosuppressive agents. Patients with acute systemic infections or non-HBV-related malignancies were also excluded. Detailed information regarding the patients observed in this study is listed according to the clinical stage of HBV-associated diseases and described under Section 3.1. Continuous variables (age, Body Mass Index, Stiffness, Total Bilirubin, Albumin, AST, ALD, GGT, Platelets, INR, creatinine, and HBV DNA were expressed as medians with ranges (min–max) and categorical variables (Gender, Hypertension, Diabetes Mellitus, Hyperlipidemia, HBeAg^+^) were presented as counts and percentages. Statistical comparisons between the three groups (CHB, CHB-LC, and CHB-HCC) were performed using the Kruskal–Wallis test. For categorical variables, the Chi-square test or Fisher’s exact test was used to compare the three tested groups. The significance level of *p* < 0.05 was considered statistically significant.

### 2.2. Serum Preparation for Nuclear Magnetic Resonance (NMR)

Serum samples were collected and stored at a temperature of −80 °C. For NMR analysis, the frozen serum samples were thawed using a water bath and were mixed thoroughly by using a vortex mixer. Then, 500 µL of each sample was transferred to an Amicon^®^ Ultra−0.5 centrifugal filter unit (10 kDa) (Milli-pore) and centrifuged for 15 min at 13,000 rpm, 4 °C for the removal of proteins from the serum samples. After the protein separation, 300 µL of the resulting serum was mixed with 300 µL of PBS buffer (pH 7.4) in a 1.5 mL tube while the sample was kept on ice. The mixture was then thoroughly mixed, and 600 µL of the sample was transferred into an NMR tube for analysis using a Bruker 600 MHz spectrometer (Bruker BioSpin GmbH, Rheinstetten, Germany).

### 2.3. ^1^H-NMR Data Processing and Multivariate Statistical Analysis

Serum ^1^H-NMR was used to analyze the level of metabolites in serum samples from patients with CHB, CHB-LC, and CHB-HCC. The raw spectral data acquired from the ^1^H-NMR experiment underwent baseline correction, reference deconvolution, metabolite assignment, and binning with Chenomx NMR Suite software (Version 8.6, Edmonton, AB, Canada). The spectral data were binned from 0.04 to 9.98 ppm with a 0.04 ppm bin width, except for the water suppression region at 4.60–5.20 ppm. The relative intensity values for metabolites of interest were calculated by the division of the area of the spectral data obtained by the area of the reference feature trimethylsilyl propanoic acid (TSP). This calculation resulted in the division of the spectrum into 235 integrated regions, covering the 0.0–10.0 ppm range. Residual water signals (δ 4.60–5.20 ppm) were excluded from further analysis. The binned dataset was imported into SIMCA-P+ (version 15.0, Umetrics, Umeå, Sweden) for further statistical analyses of the metabolites present in the serum samples. Normalization by sum followed by pareto-scaling (mean-centering and division by the square root of the standard deviation per variable) was performed to account for potential variations in sample concentration.

After data preprocessing, the statistical analysis began with a univariate one-way Analysis of Variance (ANOVA) for the assessment of significant differences in the metabolite concentration across three tested groups (CHB, CHB-LC, CHB-HCC). Then, Fisher’s Least Significant Difference (FLSD) post hoc test was applied for pairwise comparisons and the False Discovery Rate (FDR) was used to adjust the data for multiple comparisons and minimize false positives. A hierarchical cluster analysis (HCA) was used for the visualization of clustering patterns of metabolites that were statistically significant based on the ANOVA test with FDR correction and the FLSD post hoc test across the three tested groups. Then, a multivariate Orthogonal Partial Least Squares Discriminant Analysis (OPLS-DA) was applied to classify metabolic profiles according to three tested groups and for the examination of the contribution of individual identified metabolites to the clinical stage of the liver disease. The OPLS-DA model was internally validated using total explained variance (R^2^ Y) and predictive ability (Q^2^Y) values. For external validation, a permutation test was conducted for the comparison of the R^2^Y and Q^2^Y values of the original model with those from randomly permutated models for the assessment of prediction reliability. Then, the OPLS-DA-derived metabolites were ranked based on Variable Influence of Projection (VIP) scores, based on their contribution to group discrimination. Metabolites with VIP scores above 0.9 were considered as significant contributors to the group discrimination of the three clinical stages of liver disease. For the analysis of metabolite relationships across the tested groups and enriched pathways, a correlation heatmap and pathway enrichment was generated for the visualization of clustering patterns within the binned dataset using the MetaboAnalyst Software (Version 5.0, http://www.metaboanalyst.ca/, accessed on 10 March 2021), with the incorporation of interquartile range (IQR)-based data filtering. In conclusion, the identification of significant metabolites that can be used as specific biomarkers for the three clinical stages of HBV liver disease and related liver degradation was based on the following statistical approaches: (1) ANOVA with FDR correction for the determination of significant differences in metabolite concentrations across the tested groups and Fisher’s LSD post hoc test for pairwise metabolite comparisons, (2) HCA for the visualization of metabolite profile clustering and pattern recognition, (3) OPLS-DA loading plot (OPLS-LP) and VIP score ranking analysis (VIPS) for the classification and ranking of significant differential metabolites according to the clinical stages of HBV liver disease, (4) Pearson correlation analysis for explorations of metabolite relationships and patterns observed between the clinical stages of HBV liver disease, and (5) pathway enrichment analysis for the identification of metabolic pathways significantly associated with the 64 identified metabolites.

## 3. Results

### 3.1. The Clinical Characteristics of the Study Population of Patients with the Progression of HBV-Associated Liver Disease

A total of 114 patients were included in the present study. These 114 patients were grouped according to the level of progression of HBV infection from early CHB (60) to advanced CHB-LC (35) or CHB-HCC (15). All the patients were assessed as being positive for the hepatitis B surface antigen (HBeAg). The levels of aspartate transaminase (AST), alanine transaminase (ALT), gamma-glutamyl transferase (GGT), alkaline phosphatase (ALP), and international normalized ration (INR) were considered as important criteria for the characterization of CHB progression. The clinical characteristics of the patients described in the present study are summarized in Table 1.

### 3.2. The Identification of Serum Metabolites in Patients with the Progression of HBV-Associated Liver Disease

In the present study, a total of 64 metabolites were identified by using ^1^H-NMR and peak fitting with the Chenomx database after the calibration of the chemical shift with TSP. The majority of the identified metabolites were classified as primary and/or secondary metabolites, such as bile salt (glycocholate), organic acids (2-hydroxyisovalerate, 2-hydroxyvalerate, 2′-deoxyadenosine, 2-hydroxybutyrate, 3-hydroxybutyrate, 3-hydroxyisobutyrate, acetate, citrate, formate, glucuronate, glycolate, lactate, malate, pyruvate, succinate), amino acids (isoleucine, 2-aminobutyrate, glutamate, alanine, alloisoleucine, citrulline, tauine, anserine, asparagine, aspartate, serine, carnosine, cystine, glutamine, phenylalanine, histidine, homoserine, proline, lysine, ornithine, glycine, threonine, sarcosine, tryptophan, tyrosine, valine, betaine, arginine), alcohol (ethanol), hydro pyrimidines (5,6-Dihydrothymine), enzyme inhibitor (oxypurinol), sugars (cellobiose, glucose, mannose), sugar alcohols (glucitol, glycerol, mannitol, propylene glycol), amino acid derivatives (creatinine, creatine), and organic compounds (phosphate, dimethylamine (DMA), histamine, methylamine, N-nitrosodimethylamine, trimethylamine (TMA), trimethylamine-N-oxide (TMAO), carnitine, choline), as described in Supplementary Table S1.

**Figure 1:**
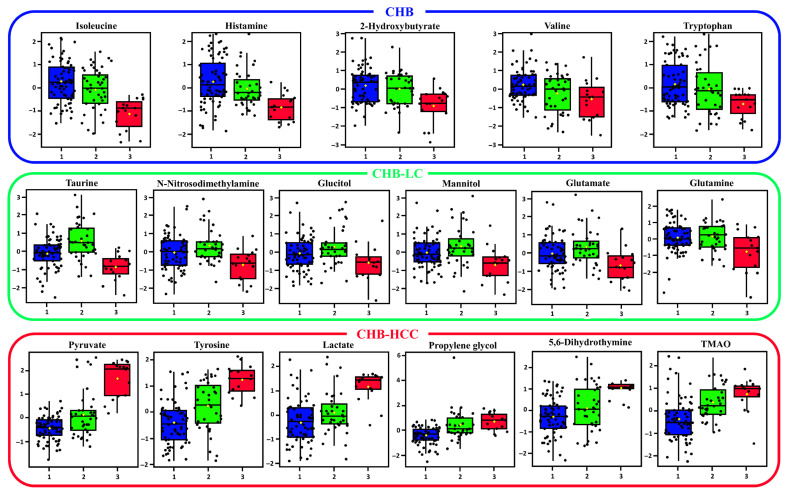
Boxplot of significantly elevated metabolites across tested groups according to one-way ANOVA with FDR correction and Fisher’s LSD test for pairwise comparison.

**Figure 2:**
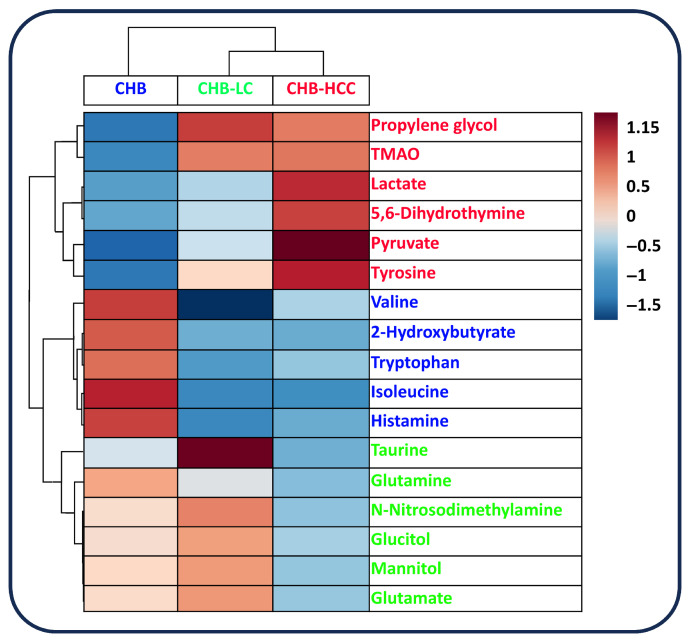
Heatmap for hierarchical clustering of these metabolites with red/blue indicating higher/lower relative concentrations.

Among the 64 metabolites, 17 metabolites were found to be statistically significant in univariate ANOVA analyses across the tested groups of patients, according to the progression of the HBV-associated liver disease (Table 2, Figure 1). These 17 metabolites (pyruvate, tyrosine, lactate, propylene glycol, isoleucine, taurine, 5,6-dihydrothymine, TMAO, histamine, 2-hydroxybutyrate, N-nitrosodimethylamine, mannitol, glutamate, valine, glutamine, tryptophan, glucitol) can be used as differential metabolites, which can help distinguish between stages of HBV-associated liver disease progression. Figure 1 depicts the relative abundance of 17 significant metabolites across the three groups, while Figure 2 represents a heatmap with the hierarchical clustering of the 17 metabolites for the visualization of their stage-specific enrichment and expression patterns. The metabolites with the highest relative concentration in each group are as follows: (1) CHB: isoleucine, histamine, 2-hyroxybutyrate, valine and tryptophan; (2) CHB-LC: taurine, N-nitrosodimethylamine, glucitol, mannitol, glutamate, and glutamine; and (3) CHB-HCC: pyruvate, tyrosine, lactate, propylene glycol, 5,6-dihydrothymine, and TMAO.

### 3.3. The Multivariate Analysis of Serum Metabolite Data from Patients with the Progression of HBV-Associated Liver Disease

**Figure 3:**
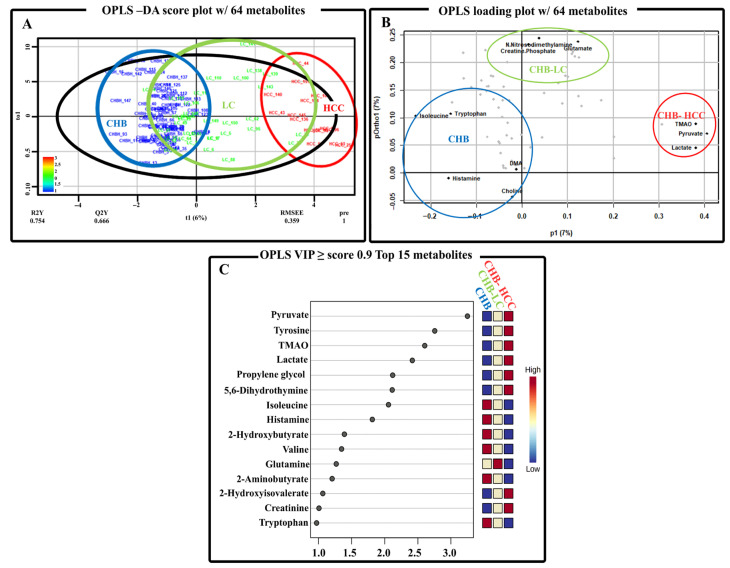
(**A**) OPLS-DA score plot of serum samples from patients with CHB, CHB-LC, and CHB-HCC. (**B**) Multivariate statistical analysis of serum metabolites across HBV-associated liver disease progression with visualization of metabolite distribution in OPLS loading plot with key metabolites as per group as follows: (1) CHB-Isoleucine, Tryptophan, DMA, Choline, Histamine, (2) CHB-LC-N-nitrosodimethylamine, Creatine, Glutamate, Phosphate, and (3) CHB-HCC-TMAO, Lactate, Pyruvate). (**C**) OPLS VIP score analysis of key discriminatory metabolites that contribute to group separation, and heatmap of relative metabolite intensities.

Based on the ^1^H-NMR data, a multivariate analysis using supervised OPLS-DA was used for the identification of differential metabolites that contributed to group discrimination and for the comparison of stage-specific metabolite profiles during HBV-associated liver disease progression (CHB, CHB-LC, CHB-HCC). The OPLS model was constructed with one predictive and two orthogonal components, which demonstrated distinct clustering and clear separation between the three groups (Supplementary Figure S1; Figure 3A). This confirms that there was a distinct shift in the metabolite profiles during HBV-associated disease progression. The OPLS loading plot was used for the identification of statistically significant metabolites that contributed to group discrimination, while VIP score analyses were used to rank metabolites based on their influence in group classification according to the stage of liver disease progression (Figure 3).

In Figure 3B, an OPLS loading plot was constructed based on the 64 metabolites identified by the ^1^H-NMR analyses. The loading plot demonstrates a distinct separation of metabolite profiles corresponding to CHB, CHB-LC, and CHB-HCC and identifies 12 key metabolites that substantially contribute to the discrimination among these disease stages. The 12 key discriminatory metabolites identified to be associated stage-specific HBV liver disease are as follows: CHB (isoleucine, histamine, tryptophan, choline, DMA), CHB-LC (N-nitrosodimethylamine, glutamate, creatine, phosphate), and CHB-HCC (TMAO, pyruvate, lactate). In parallel, the VIP score analysis (Figure 3C) identified 15 differential metabolites (VIP ≥ 0.9) that strongly influenced group discrimination between disease progression in the OPLS-DA model (Figure 3B). As depicted in Figure 3C, a heatmap exhibits the rank and relative abundance patterns of these key metabolites across the tested groups and highlights their contribution to group distinction as follows: CHB (isoleucine, histamine, 2-hydroxybutyrate, valine, 2-aminobutyrate, tryptophan,), CHB-LC (glutamine), and CHB-HCC (pyruvate, TMAO, tyrosine, lactate, propylene glycol, 5,6-dihydrothymine, 2-hydroxyisovalerate, creatinine). Among these, 12 metabolites that were significantly discriminatory in the ANOVA analyses were also found to have significantly high relative concentrations in CHB (isoleucine, histamine, tryptophan, 2-hydroxybutyrate, valine), CHB-LC (glutamine), and CHB-HCC (tyrosine, propylene glycol, 5,6-dihydrothymine, TMAO, pyruvate, lactate) (Figure 1).

In the present study, six discriminatory metabolites were consistently identified as statistically significant across all three analytical approaches—univariate analysis (ANOVA with FDR and Fisher’s LSD), multivariate OPLS loading plot, and VIP score analysis—and were designated as core metabolites associated with the progression of HBV-related liver disease. These high-confidence metabolites, elevated in CHB (isoleucine, tryptophan, histamine) and CHB-HCC (pyruvate, TMAO, lactate), are proposed as robust biomarkers for distinguishing specific stages of HBV-associated liver disease.

### 3.4. Correlated Metabolites of Six Core Discriminatory Metabolites in the Progression of HBV-Associated Liver Disease

**Figure 4:**
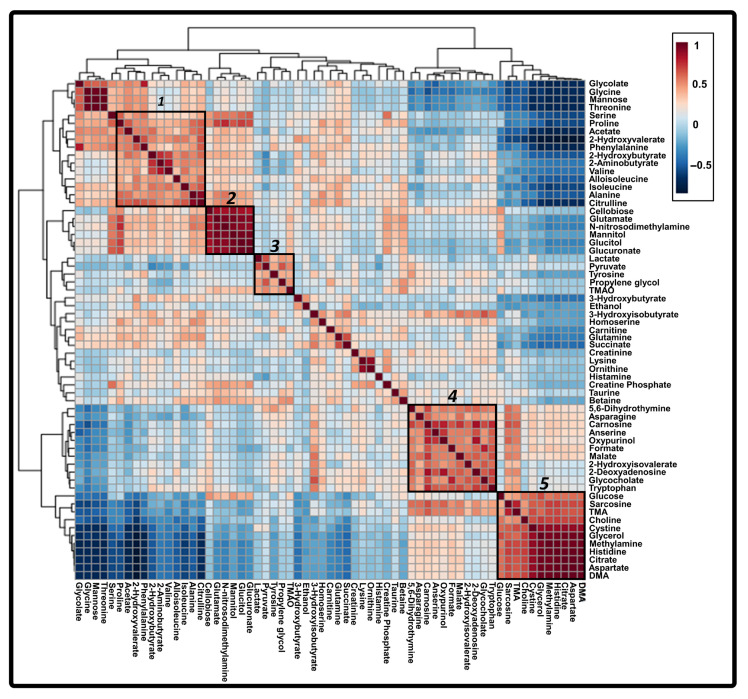
A Pearson-correlation-based clustered heatmap depicting the relationships between the 64 identified metabolites in the serum samples, with hierarchical clustering and color-coded correlation values. The numerically labelled boxes in the figure correspond to the following key metabolites–clinical stages of HBV liver disease: 1. Cluster 1-isoleucine-CHB. 2. Cluster 2-glutamate, N-Nitrosodimethylamine—CHB-LC. 3. Cluster 3-TMAO, lactate, pyruvate-CHB-HCC. 4. Cluster 4-tryptophan-CHB. 5. Cluster 5-DMA, choline-CHB.

To investigate the correlated metabolites of the six core discriminatory metabolites, all of the 64 metabolites detected in the 1H-NMR based analyses were used in conducting a supervised hierarchical clustering analysis to generate a Pearson correlation heatmap. This heatmap revealed five major metabolite clusters designated in Figure 4 and enabled the exploration of inter-metabolite relationships across HBV-associated disease stages. In cluster 1, isoleucine exhibited a strong positive correlation with amino acids (serine, proline, phenylalanine, 2-aminobutyrate, valine, alloisoleucine, alanine, citrulline) and related organic acids (acetate, 2-hydroxyvalerate, 2-hydroxybutyrate), suggesting a shift in amino acid metabolism during early-stage CHB. Cluster 4 indicated that tryptophan was coregulated with various metabolites (5,6-dihydrothymine, asparagine, carnosine, anserine, oxypurinol, formate, malate, 2-hydroxyisovalerate, 2-deoxyadenosine, and glycocholate), which could be involved in nucleotide and amino acid metabolism. Cluster 3 was clustered with TMAO, lactate, and pyruvate, which were core metabolites associated with CHB-HCC, highlighting their collective role in altered energy metabolism and the Warburg effect. These cluster-specific patterns suggest that core metabolites not only differentiate disease stages but also participate in coordinated metabolic responses during HBV-associated liver disease progression. The six core discriminatory metabolites were not included in cluster 2 and 5. Cluster 2 included mannitol, glucitol, glutamate, and N-Nitrosodimethylamine, which were all metabolites that had notably high relative abundances in the CHB-LC group, according to the univariate statistical analysis (Figure 1). For cluster 5, glucose, sarcosine, TMA, cysteine, glycerol, methylamine, histidine, citrate, and aspartate were included, along with DMA and choline, which were identified in the OPLS loading plot as being associated with CHB patients (Figure 3B).

### 3.5. Metabolic Pathways Affected by the Progression of HBV-Associated Liver Disease

**Figure 5:**
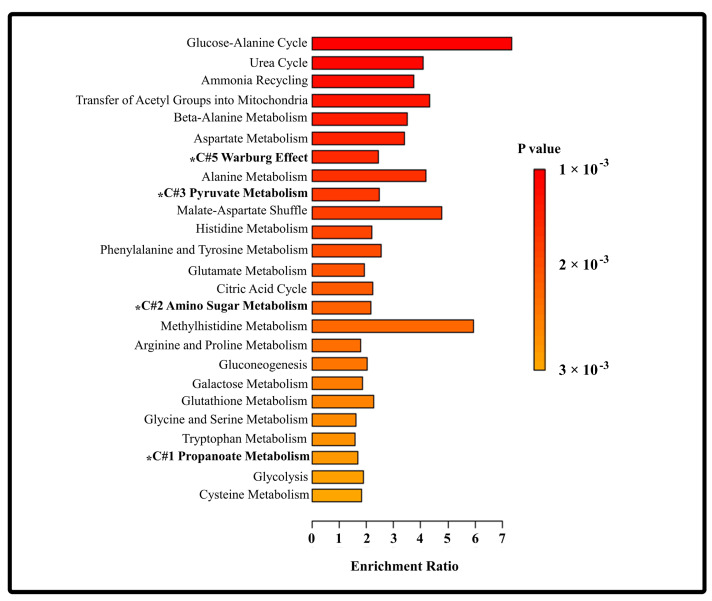
Metabolic pathway enrichment analysis of detected metabolites, with enrichment ratio and color gradient for indication of statistical significance or *p*-value. Legend: *—indicates that it is a pathway associated key differential metabolites and #—indicates cluster number according to Pearson correlation heatmap in Figure 4.

In this study, six core discriminatory metabolites that were identified consistently across univariate and multivariate statistical analyses were determined to be significantly associated with distinct clinical stages of HBV-related liver disease. To further investigate their biological relevance, a pathway enrichment analysis was performed with the 64 metabolites identified by 1H-NMR analyses (Figure 5). These metabolites were mapped to a variety of metabolic pathways involved in energy metabolism, such as gluconeogenesis, glycolysis, and the Warburg effect; organic acid metabolism; amino acid metabolism; and nitrogen waste processing. Among the 25 enriched pathways identified, the glucose–alanine cycle showed the highest enrichment score. Notably, three pathways—propanoate metabolism, pyruvate metabolism, and the Warburg effect—were strongly associated with the core metabolites enriched in specific disease stages. Elevated levels of isoleucine, histamine, and tryptophan in CHB were linked to dysregulation in propanoate metabolism, while increased concentrations of pyruvate, TMAO, and lactate in CHB-HCC corresponded to alterations in pyruvate metabolism and the Warburg effect. These findings underscore the stage-specific metabolic reprogramming that accompanies HBV-related liver disease progression and highlight the diagnostic potential of the six core metabolites as biomarkers for CHB and CHB-HCC.

## 4. Discussion

HBV-associated liver disease, such as CHB, is initially asymptomatic, which hinders early therapeutic intervention because of the lack of early diagnostic systems. Usually, HBV infection is only treated once it has progressed into late CHB and associated CHB-HCC and CHB-LC. Therefore, HBV-associated diseases can already be fatal because of the irreversible alteration and/or damage it causes to major metabolic pathways, such as the TCA cycle, amino acid, aerobic glycolysis, protein, and lipid metabolism [14,15,16]. The resulting altered metabolism also affects the quality and/or quantity of metabolites in the serum samples of afflicted patients. This process can be exploited and used for the identification of differential metabolites that can be associated with each stage of HBV-associated liver disease: CHB, CHB-LC, and CHB-HC. These differential metabolites can provide key insights into understanding the pathology of HBV-associated diseases and be used as foundation for the development of stage-specific biomarkers and/or the improvement of formulations for therapeutic treatment.

In the present study, isoleucine, histamine, and tryptophan are three core metabolites associated with CHB. Interestingly, the concentration of these metabolites continually decreased as CHB progressed into CHB-HCC (Figure 1). Histamine is a common proinflammatory factor observed in several inflammatory diseases (diabetes, obesity, colitis, arthritis) and has also been associated with liver damage caused by liver transplantation [17]. Elevated levels of histamine were associated with the advanced stages of LC [18] and HCC [19], but the levels of histamine at the early stages of CHB, CHB-LC, and CHB-HCC were not measured in these studies. Although 2-Hydroxybutyrate was not considered in the core metabolites in this study, it was statistically significant in detecting the CHB stage in the ANOVA and VIP score analyses and was identified as a differential metabolite associated with the formation of liver tumor tissue [20], hepatic fibrosis [16], cirrhosis-associated hepatocellular carcinoma [21], and alcoholic hepatitis and/or the cirrhosis-induced dysregulation of the glutathione pathway [22]. In these studies, histamine and 2-Hydroxybutyrate were associated with advanced forms of liver disease.

In the ANOVA and VIP score analyses, the levels of branched-chain amino acids (BCAAs), such as valine and isoleucine, were gradually decreased as the concentration of tyrosine, an aromatic amino acid (AAA) gradually increased with the progression of HBV-associated liver disease, from CHB to CHB-HCC (Figure 1 and Figure 3C). Interestingly, the concentration of tryptophan, another AAA, decreased as CHB progressed into CHB-HCC. The alteration to the levels of BCAAs and AAAs is because of the impaired metabolism of hepatic amino acids caused by the progression of liver diseases. The elevated levels of differential metabolites for CHB indicates its effect on the metabolism of amino acid metabolism and highlights their usefulness and prognostic significance [23]. In addition, several studies have reported that the serum levels of BCAAs (valine, leucine, and isoleucine) and a ratio of BCAAs to AAAs (tyrosine, phenylalanine, and tryptophan) gradually decreased during the progression of liver diseases [24,25]. Furthermore, several clinical and animal studies demonstrated that the supplementation of BCAAs improves the overall nutritional status and liver function in chronic liver diseases [26]. On the other hand, 2-aminobutyrate was significantly associated with CHB, according to the VIP score analysis (Figure 3C). A common intermediate in amino acid metabolic pathways is 2-aminobutyrate. In addition, 2-aminobutyrate is a key intermediate in the biosynthesis of ophthalmic acid, a metabolite that is significantly elevated in patients with cancer [27], and was reported to be a potential biomarker for hepatic glutathione (GSH) depletion due to oxidative stress [28]. In one study, GC-MS and UPLC-TOFMS identified low levels of 2-aminobutyrate as a differential metabolite in urine samples of cirrhotic patients as compared to healthy controls [29]. In another study, NMR identified low levels of 2-aminobutyrate as a differential metabolite in the blood serum of patients with HBV-associated acute-onset chronic liver failure as compared to CHB patients [30].

TMAO, pyruvate, and lactate were classified under core discriminatory metabolites for CHB-HCC patients (Figure 1, Figure 2 and Figure 3). In the correlation heatmap, cluster 3 depicts these three core metabolites as being significantly associated with propylene glycol [31,32] and tyrosine (Figure 4) [33,34,35]. In addition, the concentrations of these five metabolites, together with 5,6-dihydrothymine, are significantly elevated in the patients with CHB-HCC as compared to those with CHB or CHB-LC (Figure 1). Although propylene glycol, tyrosine, and 5,6-dihydrothymine are not core metabolites in the present study, they were statistically significant in the ANOVA and VIP score analyses for the CHB-HCC patients. To the best of our knowledge, this is the first identification and quantification of 5,6-dihydrothymine, an intermediate of thymine metabolism, in the serum of patients with the progression of HBV-associated liver disease. Several studies have identified TMAO, pyruvate, lactate, propylene glycol, and tyrosine as biomarkers for CHB-HCC [32,33,34,35,36,37]. All these metabolites have strong positive correlations with the altered metabolism of carbohydrates (pyruvate metabolism, glycolysis/gluconeogenesis, TCA cycle) and amino acids and with the biosynthesis of AAAs (Figure 5). Interestingly, the relative abundance of tyrosine increased as HBV-associated liver disease progressed from CHB to CHB-HCC (Figure 1). A similar trend was observed in comparative metabolomics studies between the tissue samples of patients with HCC-associated NAFLD and those with HCC-associated LC [33] and between the serum samples of patients with HCC and a healthy control group [34,35]. On the other hand, propylene glycol has been identified in the serums of patients with HCC and has been significantly associated with HCC development [32,38]. Propylene glycol is not an uncommon metabolite to find in human serum samples [39]. It is assumed that this is because propylene glycol is a common solvent used in several oral, intravenous, and topical pharmaceutical medications, as well as other general commodities, such as processed food, cosmetics, and toothpastes. Normally, healthy livers and kidneys metabolize propylene glycol into pyruvate, acetate, and lactate within several hours [40]. Thus, the high concentration of propylene glycol could be because of a high intake of medication, which is normal for patients with impaired liver function [32]. High concentrations of propylene glycol and associated metabolites, lactate, and pyruvate (Figure 1) also indicate that CHB-HCC is positively correlated with altered carbohydrate metabolic pathways (Figure 5). In addition, high levels of lactate and pyruvate are also indicators of enhanced aerobic glycolysis, or the Warburg effect, a typical metabolic characteristic associated with the development of cancer cells [15,16,17,18,41,42,43,44,45,46,47].

Interestingly, the elevated level of TMAO was also observed as CHB progressed into CHB-LC and CHB-HCC (Figure 1). This indicates that there was significant interaction between the metabolites derived from gut microbiome and the liver [15]. This also confirms that the liver–gut axis plays a crucial role in the progression of HBV disease [45,46,47]. TMAO is produced in the liver by the oxidation of trimethylamine (TMA) using hepatic flavin-dependent monooxygenase (FMO). TMA is a metabolite derived from the metabolism of choline, carnitine, and betaine by the gut microbiome. Several studies indicate that the elevated serum level of TMAO is also correlated with gut microbial dysbiosis and an increased risk of the development of cardiovascular, neurodegenerative, and liver diseases and cancers [45,46,47]. Thus, TMAO is already considered as a potential biomarker and target for the development of novel diagnostic and therapeutic technologies [45,47]. However, in terms of liver disease, TMAO is mostly correlated with NAFLD and its associated hepatic steatosis, metabolic dysregulation, and HCC [48]. For example, the elevated level of TMAO is accompanied by tissue inflammation, the decreased production of bile-acid-derived enzymes, and the development of hepatic insulin resistance in patients with NAFLD disease [49]. For example, the serum level of TMAO is higher in NAFLD patients, which indicates a strong correlation between the TMAO level and the severity of hepatic steatosis [50]. In another study, the elevated TMAO level is related to NASH in obese patients with type 2 diabetes [51]. TMAO can exacerbate glucose intolerance, promote the expression of proinflammatory factors, and decrease the levels of anti-inflammatory cytokine, IL-10, in adipose tissue [52]. In addition, the metabolic change in the serum levels of liver osmolytes, such as taurine and TMAO, observed in this study is an indication of metabolic adaptation to the oxidative stress caused by HBV-associated liver disease (Figure 1). However, it remains unknown whether serum TMAO levels may serve as a biomarker for the prognosis of NAFLD and the associated metabolic dysregulation [53,54]. In terms of hepatitis-virus-related liver disease, high levels of TMAO have been measured in the urine samples of patients with hepatitis B- and hepatitis C-associated HCC [55,56].

## 5. Conclusions

In conclusion, this study employed ^1^H-NMR-based metabolomic profiling to identify and quantify the key differential metabolites associated with stage-specific metabolic alterations during the progression of HBV-associated liver disease, including CHB, CHB-LC, and CHB-HCC. Distinct serum metabolic profiles were observed across the three disease stages. Among the 64 metabolites identified, six were consistently significant across all the statistical analyses—univariate ANOVA with FDR and Fisher’s LSD, an OPLS loading plot, and VIP score analysis—and were designated as core discriminatory metabolites. These included isoleucine, tryptophan, and histamine, which were elevated in CHB, and pyruvate, TMAO, and lactate, which were elevated in CHB-HCC. Subsequent correlation and pathway enrichment analyses revealed that these metabolites were grouped into specific clusters associated with distinct metabolic pathways, which were amino acid metabolism, propanoate metabolism, pyruvate metabolism, and the Warburg effect. These findings suggest that the six core metabolites have strong potential as stage-specific biomarkers for monitoring the progression of HBV-related liver disease. The ^1^H-NMR approach to analyzing metabolomic profiles for the characterization of CHB progression conducted in this study provides the basis for future research and contributes to the development of advanced and precise prediction models. The clinical application of metabolomics is still premature, and it remains a challenging technology to establish since precise and cost-effective systems for the analysis of metabolomes have yet to be developed. The observations reported in this study confirm that there is an important inter-relationship between the progression of HBV-associated liver disease and the metabolic pathways and metabolites related to the gut microbiome–liver axis. However, the role and effect of differential metabolites and altered pathways on the stages of HBV-associated liver disease progression are still not fully understood. Although this study contributed to further understanding this problem, this study is limited by the number of subjects. Further studies with the use of multiple metabolomics techniques/approaches and clinical studies are needed for the in-depth comparison and confirmation of the findings described in this study and for the elucidation of the molecular pathogenesis of HBV-associated liver disease.

## Figures and Tables

**Table 1:** The baseline characteristics of the patients, classified according to the progression of HBV-associated liver disease (CHB, CHB-LC, CHB-HCC). Continuous variables are expressed as means and standard deviations, and categorical variables are expressed as numbers and percentages.

Criteria	CHB (*n* = 64)	CHB-LC (*n* = 35)	CHB-HCC (*n* = 15)	*p*-Value
Age (years)	49 (23–75)	51 (32–74)	51 (33–83)	0.6551
Gender (Male)	48 (75.0)	24 (68.6)	14 (93.3)	0.2661
Hypertension	9 (14.1)	8 (22.9)	3 (20.0)	0.1889
Diabetes Mellitus	10 (15.6)	4 (11.4)	2 (13.3)	0.3528
Hyperlipidemia	5 (7.8)	2(5.7)	1 (6.6)	0.5002
Body Mass Index (kg/m^2^)	24.8 (18.1–44.4)	24.0 (18.1–29.0)	23.1 (16.5–28.4)	0.1778
Stiffness (kPa)	5.85 (3.3–9.9)	12.4 (14.7–46.4)	13.0 (6.0–33.8)	<0.0001
Total Bilirubin (mg/dL)	0.75 (0.2–12.7)	0.95 (0.2–4.9)	1.3 (0.4–19.9)	0.02144
Albumin (mg/dL)	4.2 (2.9–4.8)	4.0 (2.2–5.2)	2.9 (2.1–4.3)	0.002864
AST (IU/dL)	54 (12–2290)	52.5 (20–1430)	90 (51–369)	0.01426
ALT (IU/dL)	84 (4–2140)	42.5 (15–1402)	52 (11–759)	0.2416
GGT (mg/dL)	37 (11–767)	53.5 (9–889)	163 (13–535)	0.02091
Platelet (10^3^/μL)	206 (106–465)	167 (22–300)	159 (64–360)	0.007749
INR	1.06 (0.93–1.37)	1.16 (0.93–1.96)	1.24 (0.96–2.27)	0.005469
Creatinine (mg/dL)	0.75 (0.46–4.29)	0.72 (0.50–3.52)	0.90 (0.36–5.11)	0.3903
HBeAg (+)	31 (48.4)	15 (42.9)	5 (33.3)	0.5657
HBV DNA (IU/L)	2 × 10^6^ (0–1 × 10^9^)	7 × 10^5^ (0–2 × 10^8^)	1 × 10^6^ (4.0 × 10^1^–1 × 10^7^)	0.2458

**Table 2:** Identification of 17 significant metabolites according to statistical analyses (ANOVA, False Discovery Rate, Fisher’s LSD test).

Metabolites	*f*. Value	*p* Value	FDR	Fisher’s LSD
Pyruvate	52.265	9.27 × 10^−17^	5.84 × 10^−15^	2-1; 3-1; 3-2
Tyrosine	24.954	1.09 × 10^−9^	3.43 × 10^−18^	2-1; 3-1; 3-2
Lactate	17.216	3.02 × 10^−7^	6.35 × 10^−6^	2-1; 3-1; 3-2
Propylene glycol	15.883	8.46 × 10^−7^	1.33 × 10^−5^	2-1; 3-1
Isoleucine	15.075	1.59 × 10^−6^	2.01 × 10^−5^	1-3; 2-3
Taurine	14.629	2.27 × 10^−6^	2.38 × 10^−5^	2-1; 1-3; 2-3
TMAO	13.295	6.60 × 10^−6^	5.19 × 10^−5^	2-1; 3-1
Histamine	8.857	0.00026851	0.0018796	1-3; 2-3
2-Hydroxybutyrate	8.0749	0.00052966	0.0033368	1-3; 2-3
N-Nitrosodimethylamine	6.6211	0.0019149	0.010967	1-3; 2-3
Mannitol	5.8654	0.0037796	0.019843	1-3; 2-3
Glutamate	5.7421	0.0042263	0.020481	1-3; 2-3
Valine	5.3763	0.0058952	0.025792	1-2; 1-3
Glutamine	5.3315	0.006141	0.025792	1-3; 2-3
Tryptophan	4.7257	0.010707	0.042159	1-3; 2-3
Glucitol	4.6325	0.011668	0.043241	2-3
5,6-Dihydrothymine	13.607	5.13 × 10^−6^	4.62 × 10^−5^	2-1; 3-1; 3-2

## Data Availability

The data presented in this study are available upon request from the corresponding author, due to ethical restrictions.

## Supplementary Materials

The following supporting information can be downloaded at: https://www.mdpi.com/article/10.3390/metabo15080504/s1. Figure S1: The OPLS-DA model of the metabolites in the serum of patients with different stages of HBV-associated liver disease (CHB, CHB-LC, CHB-HCC) with the following information: (A) a bar plot of the predicted values for the OPLS model; (B) R2Y and Q2Y values according to the permutation test; (C) a diagnostic plot with outlier samples; and (D) an OPLS-DA score plot of the serum samples from patients with CHB, CHB-LC, and CHB-HCC. Table S1: Correlation coefficient values for identified serum metabolites in patients with HBV.
