# Supplementary material for: Metabolomic Profiling of Hepatitis B-Associated Liver Disease Progression: Chronic Hepatitis B, Cirrhosis, and Hepatocellular Carcinoma
Source: Metabolites. 2025 Jul 29;15(8):504. doi: 10.3390/metabo15080504 (PMC12388518; doi:10.3390/metabo15080504)

Supplementary Information

Article

Metabolomic Profiling of Hepatitis B-Associated Liver Disease Progression: Chronic Hepatitis B, Cirrhosis, and Hepatocellular Carcinoma

Junsang Oh[1,2,†], Kei-Anne Garcia Baritugo[1,2,†], Jayoung Kim[1,3], Gyubin Park[4], Ki Jun Han[5], Sangheun Lee[5,*], and Gi-Ho Sung,[1,2,*]

[1] Biomedical Institute of Mycological Resource, International St. Mary's Hospital and College of Medicine, Catholic Kwandong University, Incheon 22711, Republic of Korea; lordjs05@gmail.com (J.O.); 1204keianne@gmail.com (K.-A.G.B.); lmkjy7@gmail.com (J.K.); foreverglee@ish.ac.kr (S.L.); sung97330@gmail.com (G.-H.S.)

[2] Department of Convergence Science, College of Medicine, Catholic Kwandong University, Gangneung-si 25601, Gang-won-do, Republic of Korea; sung97330@gmail.com (G.-H.S.)

[3] Department of Laboratory Medicine, International St. Mary's Hospital and College of Medicine, Catholic Kwandong University, Incheon 22711, Republic of Korea; lmkjy7@gmail.com (J.K)

[4] Department of Biomedical Science, Graduate School, Catholic Kwandong University, Gangneung-si 25601, Gang-won-do, Republic of Korea; minhyean1004@gmail.con (G.P.)

[5] Department of Internal Medicine, International St. Mary's Hospital, College of Medicine, Catholic Kwandong University, Incheon 22711, Republic of Korea; 545818@ish.ac.kr (K.J.H.); foreverglee@ish.ac.kr (S.L.)

† Junsang Oh and Kei-Anne Garcia Baritugo share primary co-authorship

* Correspondence: foreverglee@ish.ac.kr (S.L.); sung97330@gmail.com (G.-H.S.)

Supplementary Figure 1: OPLS-DA model of metabolites in serum of patients with different stages of HBV-associated liver disease (CHB, CHB-LC, CHB-HCC) with following information: (A) Bar plot of predicted values for OPLS model; (B) R2Y and Q2Y values according to permutation test; (C) Diagnostic plot with outlier samples; and (D) OPLS-DA score plot of serum samples from patients with CHB, CHB-LC and CHB-HCC.

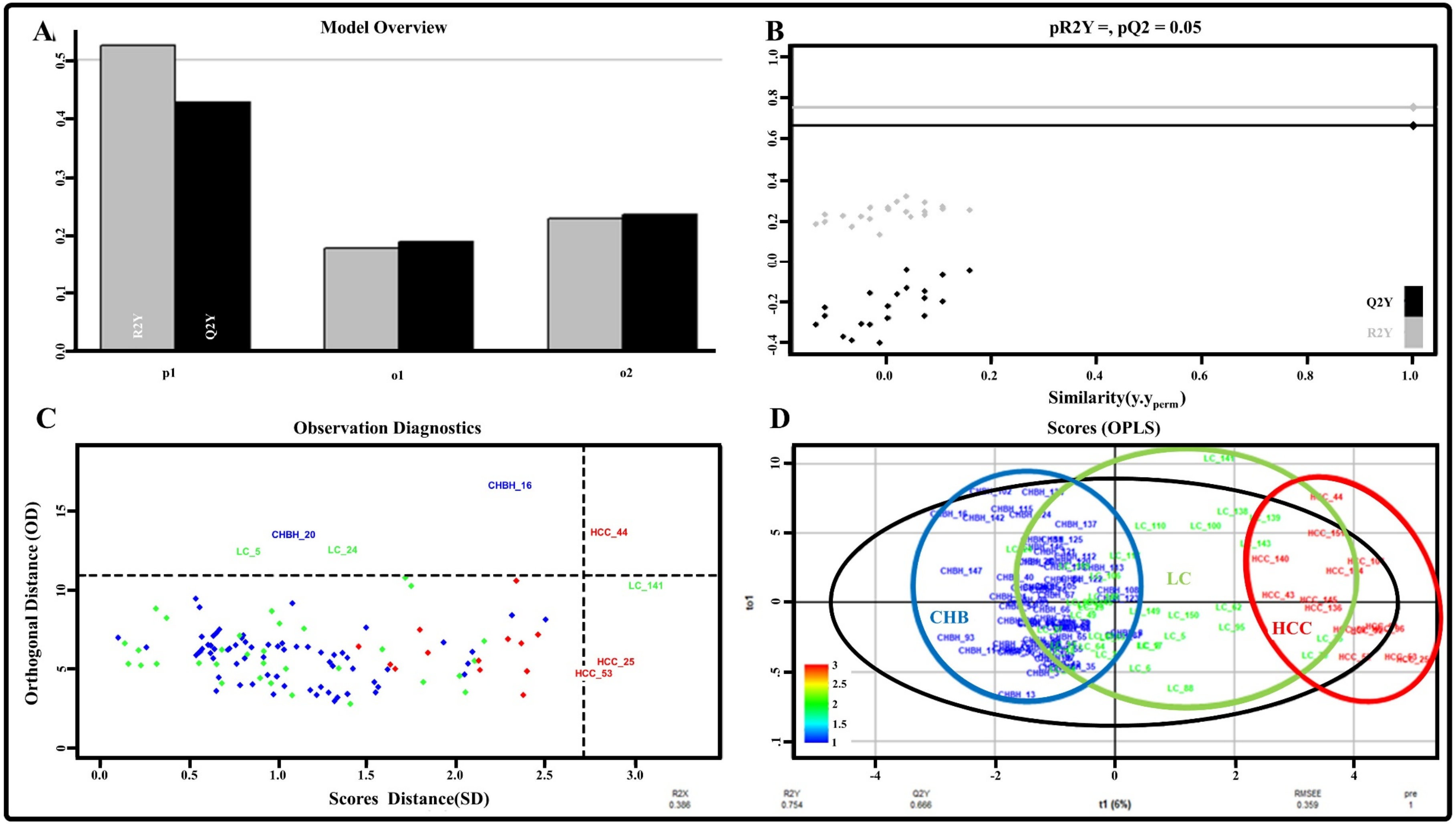

Supplement: Supplementary file 1 [file metabolites-15-00504-s001.zip › metabolites-3693030-supplementary (5).pdf]
